# Repertory of the Back

**Published:** 1899-11

**Authors:** E. H. Wilsey

**Affiliations:** Parkersburg, W. Va.


### REPERTORY OF THE BACK.

E. H. Wilsey, M. D., Parkersburg, W. Va.

(Continued from Oct. number, page 472.)

### COCCYX.

**Abdomen**, tearing pain in coccyx, better by pressing hands against abdomen, *Merc.*

**Aching**, rheumatic drawing and aching from loins to coccyx, *Carbo-v.*

— excessive aching and neuralgia of coccyx, Fluor-ac.

— pain and aching soreness, pressure, tearing and shooting in os coccyx, *Calc-ph.*

— pushing, aching, or at times pinching pain in coccyx, *Zinc.*

— coccyx seems elongated, sensitive to pressure and aching, could not sit except on a cushion, Xanthox.

**Across**, sticking across coccyx before stool, Sep.

**Around**, heat around coccyx, *Calc-c.*

**Below**, ulcers below coccyx, *Peonia.*

— pressive pain as from soreness below coccyx, Carbo-v.

**Bent back**, sudden violent concussive tearing, stitching pain in coccygeal region as if spine were bent back, Mag-c.

**Bladder**, jerks in coccyx toward bladder, compelling her to urinate, Carbo-a.

— pain in os coccyx, sore to touch, on pressure pain extends through to bladder, worse by motion and exercise, Fluor-ac.

**Blow**, sensation of numbness in coccyx, as after a blow, while sitting, *Plat.*

**Bruised** pain in coccyx extending to sacrum, *Ruta.*

— coccyx feels bruised, *Carbo-a.*

— drawing bruised pain in coccyx, *Carbo-a.*

— or darting in os coccyx, *Caustic.*

— violent pain as from a bruise in small of back and in coccyx when touched, Alum.

### COCCYX.

**Bruised** pain in coccyx while sitting still, especially in slumber, Amm-m.

— straining pain as from a bruise on coccyx, Carbo-a.

— feeling in coccyx, Aloe.

— pain in coccyx when sitting, worse when sleeping, *Amm-m.*

— pain as from a bruise in coccyx, Caustic.

— pain in coccyx all day, *Sul.*

— burning pain in coccyx preventing sitting, worse from touch, Cist-can.

**Burning** pain which on touching the place becomes burning pain, Carbo-a.

— drawing up the back from coccyx, apparently under the skin, Mur-ac.

— pressure in coccygeal region; worse from any attempt to sit down in evening, *Apis.*

— feels burning when touched, *Carbo-an.*

**Bubbling**, pressure above anus in coccygeal region at 9 A. M., Valer.

**Clucking** in coccyx periodically, Aloe.

**Coccyodynia**, *Apis, Calc-ph., Canth, Con., Fluor-ac., Gambog., Hyper., Kali-c., Lach., Merc., Phos., Rhus., Ruta., Sepia., Zinc., Kreos.*, Act-rac., Æsc-h., Bell., Cann-s., Lactu-v., Cicuta., Paris., Petrol., Phos-ac., Plat., Sil., Thuja.

— coming on for the first time during the catamenia after parturition, Cicuta.

— with milky leucorrhœa, *Kreos.*

— after confinement, Tarant.

— with excessive aching, especially in rheumatic persons, *Fluor-a.*

— coccyx hurts after riding, Sil.

**Coxalgia**, hip-joint feels as if femur were fastened to os innominatum with iron claws, accompanied by pains which dart periodically from sacro-lumbalis muscle into the thigh, *Coloc.*

**Constant** pain in sacrum and coccyx, *Lach.*

### COCCYX.

**Cramps,** painful in coccyx, *Grat.*

**Crawling,** violent itching and crawling on the coccyx, so that he cannot bear it without scratching, Borax.

**Creeping** in sacrum, extends to coccyx, Aloe.

**Cutting** pain in abdomen, and simultaneous a dull shooting pressure in coccyx, Phos-a.

**Darting** in coccyx, *Caustic.*

**Diarrhœic** stools; aggravated pain in coccyx, three times daily, *Euphorb.*

**Dragging,** sensation as if a heavy load was hanging on end of coccyx, dragging downward all the time, *Ant-t.*

— and sometimes pinching in coccyx, Zinc.

— bruised pain in coccyx, *Carbo-a.*

**Drawing** dull pain in region of coccyx, *Caustic.*

— and rheumatic aching from loins to coccyx, *Carbo-v.*

— heaviness in os coccyx, extending along pelvis, with painful drawing as before menses, Arg-n.

— dull in coccyx in evening, Graph.

— pain along coccyx to rectum and vagina, where a spasmodic pain is felt (contractive), *Kreos.*

— sticking stitches in coccyx, Rhus.

— and burning up the back, apparently under the skin, Mur-ac.

— and tearing pinching in coccyx, *Calc-c.*

— pain in coccyx prevents erect position after long sitting, Thuja.

— in coccyx during menses, *Cic.*

**Elongated**, coccyx seems elongated, sensitive to pressure and aching; could not sit except on a cushion, Xanthox.

**Evening,** in bed a pricking itching in region of coccyx, Carbo-v.

**Fall,** inability to walk or stoop after a fall on coccyx with violent pain, *Hyper.*

— pain in os coccyx as from a fall, *Kali-iod.*

**Gnawing** in os coccyx, *Kali-c.*

— itching gnawing above the rectum on coccyx, Phos-ac.

— in coccyx repeated, *Gambog.*

### COCCYX.

**Gnawing** in coccyx in evening; better by stretching, Alum.

**Heat** around coccyx, *Calc-c.*

**Heavy** weight in coccyx, Ant-c.

— sensation as of a heavy load hanging on the end of coccyx, dragging downward all the time, *Ant-t.*

**Heaviness** in os coccyx, extending along pelvis, with painful drawing as before menses, Arg-n.

**Itching** stitch in coccyx, Phos-a.

— in coccyx (when standing), Vera-a.

— on the coccyx, Alum., Spig.

— on left side of coccyx an itching corrosion, Agari.

— stitches on coccyx where before there was itching, Amm-c.

— violent itching and crawling on coccyx, so that he cannot bear it without scratching, Borax.

— stitching in coccyx when sitting, Dros.

— gnawing on coccyx above rectum, Phos-a.

— intolerable itching at top of os coccyx; must scratch until parts become raw and sore, Bov.

— a pricking itching in os coccyx evening in bed, Carbo-v.

— of coccyx, extending upward; worse at night from heat of bed and rubbing or scratching, better from cold water and pinching parts, *Petrol.*

— violently in coccygeal region, the part becoming moist with scurfy formations, *Graph.*

**Jerking** in coccyx, with dysmenorrhœa, *Cic.*

— tearing and jerking in os coccyx, *Cic.*

**Jerks** in coccyx towards bladder, compelling her to urinate, Carbo-a.

**Lancination** in coccyx, *Canth.*

**Menses,** heaviness in os coccyx, extending along pelvis, with painful drawing as before menses, Arg-n.

**Menstruation,** neuralgia of coccyx during menstruation, Cic.

**Micturition,** pain in coccyx during micturition, Graph.

**Needles,** a very painful stitch through the rectum and anus, starting from the coccyx as with a hot needle, Carbo-v.

COCCYX.

**Neuralgia** of coccyx; worse by rising up; must sit perfectly still, *Lach.*
— os coccyx, *Carbo-a.*
— of coccyx, especially when driving in carriage, Nux-m.
— of coccyx during menstruation, Cic.
— of coccyx, excessive aching, Fluor-ac.
**Numbness** in sacrum and coccyx, Plat.
— sensation of numbness in coccyx as after a blow while sitting, *Plat.*
**Pain** in coccyx, a quick, penetrating pain, Mag-c.
— in coccyx while sitting, *Kali-b.*
— in sacrum and coccyx, Iod., Med.
— in left side near coccyx, in bone as if pressed forcibly by a hard body, Can-s.
— violent pain as from a bruise in small of back and coccyx when touched, Alum.
— bruised pain in coccyx while sitting still, especially in slumber, Amm-m.
— in os coccyx, Mur-ac.
— tearing in coccyx; better by pressing hand against abdomen, *Merc.*
— in coccyx at its junction with sacrum, sometimes in lower sacral vertebræ, Syph.
— of coccyx as if in the bone; worse on rising from sitting, and beginning to move, *Euphorb.*
**Palpitation,** feels it in os coccyx, Agari.
**Paralytic,** heaviness in os coccyx, with paralytic sensation preventing long sitting and obliging him while walking to stretch dorsal spine, Arg-n.
**Piercing,** sudden pain in coccyx, Mag-c.
**Pinching**, drawing, and tearing pinching in coccyx, *Calc-c.*
— dragging and sometimes pinching in coccyx, Zinc.
— straining, pressing, and sometimes pinching pain in coccyx, Zinc.
— pushing, aching, or at times pinching pains in coccyx, Zinc.

### COCCYX.

**Pressing**, after riding in morning a heavy pressing pain extending from point of os coccyx to anus, Ars-sul-rub.

**Pressure**, stinging in os coccyx which is painful to pressure, Sil.

— pain in os coccyx, sore to touch on pressure, pain extends through to bladder, worse by motion and exercise, Fluor-ac.

— a bubbling pressure above anus in coccygeal region at 9 A. M., Valer.

— burning pressure in coccygeal region, worse from any attempt to sit down in evening, *Apis*.

— as from a dull instrument in left side of sacrum above coccyx, Mosch.

—coccyx seems elongated and sensitive to pressure, and could not sit except on a cushion, Xanthox.

— acute pressure in coccyx in evening, Kali-b.

— as with a sharp point in coccyx, *Can-s*.

**Pressive**, sore pain beneath coccyx, *Carbo-v*.

— pain as from soreness below coccyx, Carbo-v.

— pain in coccyx and sacrum, which increases and diminishes, Iod.

— straining, pressive, and at times pinching pains in coccyx, Zinc.

**Pricking**, a pricking itching in region of coccyx, evening in bed, Carbo-v.

**Pulling**, sensation of pulling upward from tip of coccyx, Lil-tig.

**Pulsating** stitching in os coccygis when sitting, and with stitches between scapulæ, Paris-q.

**Raw**, intolerable itching in tip of os coccyx, must scratch until parts become raw and sore, Bov.

**Rheumatic** drawing and aching from loins to coccyx, *Carbo-v*.

**Riding**, coccyx hurts after riding, Sil.

**Rising**, painfulness of coccyx as if in the bone, worse on rising from sitting and beginning to move, *Euphorb*.

**Scabby**, elevated scabby spots on coccyx above fissure of nates, Sil.

### COCCYX.

**Scratching**, violent itching on coccyx so that he cannot bear it without scratching, Borax.

**Sensitiveness**, and pain in coccyx while sitting, *Petrol.*

— to touch (coccyx) *Euphorb.*

**Sharp** stitches in coccyx and on left side near sacrum, Agn-c.

— pain in coccyx as if an abscess were forming, Raphan.

— pain in os coccyx feeling as sitting on something sharp, *Lach.*

— pressure as with a sharp point in coccyx, *Can-s.*

**Shooting** pain and aching soreness and pressure, tearing and shooting in os coccyx, *Calc-p.*

— cutting pain in abdomen and simultaneous dull shooting pressure in coccyx, Phos-ac.

**Sitting**, pain like that of subcutaneous ulceration, worse by sitting or lying, *Carbo-a.*

— pain in coccyx every evening while sitting, *Castor-eq.*

— pain as if sitting on something sharp, pain in os coccyx when sitting, *Lach.*

— sensation of numbness in coccyx as after a blow, while sitting, *Plat.*

— pain and sensitiveness in coccyx while sitting, *Petrol.*

— tearing in coccyx while sitting, Paris-q.

— bruised pain in coccyx while sitting still, especially in slumber, Amm-m.

— pain in os coccyx, worse from walking or touch and after rising, after long sitting, *Kali-b.*

— pain in coccyx while sitting, *Kali-b.*

— painful uneasiness in coccyx, better by standing, worse by slightest movement sitting or lying on bed, or by least pressure, Tarant.

— painful in sacrum and coccyx and thighs, which after sitting long prevented his standing erect, Thuja.

**Stiffness**, great weariness and stiffness in sacrum and coccyx in evening, Petrol.

**Soreness** of os coccyx, Ars-s-r.

— pressive pain as from soreness below the coccyx, Carbo-v.

COCCYX.

**Soreness** and aching pressure, tearing and shooting pain in the os coccyx, *Calc-p.*

**Sore** moist places on os coccyx, Arum-tr.

— intolerable itching at top of os coccyx, must scratch until parts become raw and sore, Bov.

**Sticking,** drawing stitches in coccyx, Rhus.

— in coccyx, Colch.

— across coccyx before stool, Sep.

**Stiffness** in coccyx extending up spine, Ars.

**Stinging** in coccyx preceded by itching, Amm-c.

— in os coccyx which is painful to pressure, Sil.

**Stitch,** a very painful stitch through the rectum and anus starting from the coccyx as with a hot needle, Carbo-v.

— an itching stitch on coccyx, Phos-a.

— itching stitch in coccyx when sitting, Dros.

— in coccyx where before there was itching, Amm-c.

— acute sharp stitches in coccyx and on left side near coccyx and sacrum, Agn-c.

— violent stitches between coccyx and anus, Thuja.

— drawing sticking stitches in coccyx, Rhus.

— hot stitch extending from coccyx through rectum and anus, Carb-v.

— with tearing in os coccyx, Canth.

— sudden violent concussive tearing, stitching pain in coccygeal region as if spine was bent back, *Mag-c.*

**Stool**, wrenching pain in coccyx after stool, Grat.

**Straining** pain as from a bruise in coccyx, Carbo-a.

— pressure, at times a pinching pain in os coccyx, Zinc.

**Stretching**, gnawing pain in coccyx, in evening, better by stretching, Alum.

**Tearing** from os coccyx into middle of left thigh in the bone, evening, with piercing, Nat-s.

— in coccyx when sitting, Paris-q.

— pain in coccyx, better by pressure of hands against abdomen, *Merc.*

### COCCYX.

**Tearing** and jerking in os coccyx, *Cic.*
— drawing and pinching in coccyx, *Calc-c.*
— pain and aching soreness, pressure tearing and shooting in os coccyx, *Calc-p.*
**Touched,** coccyx pains when touched as if an ulcer was there, Phos.
— coccyx sensitive when touched, *Euphorb.*
— pains in os coccyx; worse by walking or being touched and after rising from long sitting, *Kali-b.*
**Twitches** at point of coccyx in morning, Alum.
**Ulcer,** coccyx pains when touched as if an ulcer was there, Phos.
— below coccyx, *Peonia.*
**Ulcerated,** pain in coccyx as if ulcerated, hindering motion, followed by painful stiff neck, *Phos.*
**Urinating,** while urinating a pain in os coccyx, *Graph.*
**Weariness** from coccyx upward, Brach.
— great weariness and stiffness in small of back and coccyx in evening, Petrol.

